# The Social Evil

**Published:** 1867-03

**Authors:** 


ARTICLE XVI.

### THE SOCIAL EVIL. EXTENT OF PROSTITUTION AND ITS CONSEQUENCES IN NEW YORK CITY. PROPOSED LEGAL MEASURES TO MITIGATE ITS EVILS.

The alarming prevalence of licentiousness, and the diseases resulting from it, in all the large cities of our country, is beginning to attract the serious attention of legislators as well as moralists. The subject is certainly one of the very highest importance, and cannot receive too careful a consideration by the medical profession. To aid in attracting attention, as well as in disseminating important facts, we copy entire the following report made by the medical members of the Metropolitan Board of Health of New York.

The facts presented will apply with full force to Chicago and almost every other large city in our country.—[Ed.]

The Board of Health met at 3 o'clock yesterday.

The report of the Committee on Prostitution was called for. The following is a copy:

*To the Metropolitan Board of Health:*

The Sanitary Committee, to whom was referred the resolution offered in assembly by Mr. Jacobs, viz.:

"*Resolved*, That the Board of Metropolitan Police, and the Metropolitan Board of Health, be requested to furnish to the House, at their earliest convenience, their opinion as to the necessity and the probable result of legislation looking to the more thorough restriction of prostitution in New York," beg leave, respectfully, to report that their experience as physicians had rendered them familiar with the extent to which prostitution is carried, and with the diseases arising from the diseases which emanate from it. Private practice and attendance at the dispensaries and hospitals show that these diseases are far-spread, and are of the most deplorable character.

High and low, rich and poor, are affected by venereal diseases, and no class has a monopoly of virtue. If it were so, the moral world before this would have exterminated the immoral. Prostitution has always existed in all large communities, and will continue to exist until the present order of society shall be changed, or until the golden age of virtue, which poets have foretold, and the wise and good anticipated, shall have come.

The actual and important fact is that venereal disease is sapping the strength of the people. Husbands give it to their wives and mothers give it to their children, and when it has once entered the constitution no one can tell whether it ever will or can be eradicated. Every part of the body is invaded, the skin, brain, eyes, throat, nose, lungs, and bones. The surface of the body becomes covered with sores, and with scars which endure forever afterwards. The blood itself is poisoned, and loses its red globules, which were intended to give vivacity to the brain and strength and vigor to the muscles. The poor victim of syphilis is racked with pains, permanently debilitated and incapable of hard work, and if his means are insufficient for his support must seek refuge in the hospitals or almshouses. Death is often his best friend, and if it comes early he may be saved from a lingering consumption, or some of the varied forms of scrofulous disease.

The picture is not overdrawn. The simple facts as first stated are common and beneath the truth, yet there are some persons who, from the superior excellence of their constitutions, or from using medicines early and sedulously, escapes the severe penalties of the disease. But no one can tell in advance who these favored individuals are to be, for, with all the external signs of flourishing health, there often exists some vice of constitution which syphilis will find out, develop, and render incurable.

Innocent wives get the disease from depraved husbands, and go through the same stages of suffering, broken health and premature decay. Their children are born dead or diseased, wrinkled, covered with eruptions, and, if alive, affected with a wheezing cough and catarrh. It would be a fortunate event if their lives could always terminate before they had grown to mature age, and before they were old enough to generate a race of decrepid and feeble children, to become burthens to themselves and to society.

Syphilis is so invidious that a person attacked with it never knows with certainty that he is cured. A little of the poison left uneradicated may taint again his whole system.

Your committee have known repeated instances where young men who (having once had the disease and believing themselves well) have ventured to marry, have generated diseased children, and these children, *in utero*, have given the disease to their mothers. This, too, after a period of six or eight years had elapsed since the disease was contracted, during which time these young men had led virtuous lives.

Physical pain and the ruined health of wife and children, perhaps their death, or the loss forever of the hope of offspring may seem punishment enough. But to these must be added the consciousness of guilt. Their sins will rise from the buried past, and like reproving ghosts haunt their souls forever with remorse and shame. No imagined suffering can transcend misery like this.

No one knows what the young man is who proposes to marry his daughter. He may possess a good character and the noblest qualities, and yet this daughter may give birth to diseased children, and die of the diseases which prostitutes have generated and disseminated. These facts must bring the subject home to every individual, and all ought to resolve that such diseases shall not continue to exist, if, in God's good providence they can be eradicated. The question, moreover, is not a private one, but of great political importance, for the health of the citizen is the wealth and power of the state. The laboring population are ruined in health, and made dependent upon the public charities.

During the late rebellion, the soldiers of our army were at various times incapacitated from engaging in active military operations by the prevalence among them of venereal diseases.

It would have been a profitable study to collate statistics to show the extent to which these diseases prevail in the public hospitals, dispensaries, and also in private practice. We have called for this information upon these establishments, but there has been so great delay that we cannot at the present time wait for full returns. Especially we regret that nothing of consequence has been obtained from the public institutions under the charge of the Commissioner of Public Charities and Corrections, for these are the chief resort for those afflicted with syphilitic complaints.

From Dr. Frank P. Foster, of the New York Dispensary, we have been favored with a full report. He states that the whole number treated for all complaints amounted in the year 1866 to 36,162. Of these 525 were of gonorrhœa, 11 gleet, 4 balanitis, 57 of gonorrhœal epididymitis, 467 of primary venereal sore (chancre and chancroid,) 52 of venereal bubo, 2 condyglomata, 1,033 constitutional syphilis, including 11 cases of congenital syphilis. Dr. Foster says that 22 had been registered as having diseases that were the result of venereal diseases; but it must be confessed that this statement conveys but a poor idea of the real state of the case; as first, most of the cases of these diseases have been registered simply as secondary syphilis; and second, of the 2,666 cases registered simply chronic rheumatism, and of the 135 registered as struma, it may reasonably be presumed that very many (perhaps nearly one-half) were of syphilitic origin.

In the Demilt Dispensary, Dr. Cummings has reported 176 cases of primary venereal disease, and 147 cases of constitutional veneral disease, making a total of 323.

From Randall's Island Nursery, Dr. Henry A. Whittlesey writes that "the patients treated are children between the ages of 10 and 12 years. We have, therefore, only hereditary syphilis under observation, generally modified and marked by previous treatment, rendering it impossible to give statistics of value. Of the diseases of the eyes, skin, bones, joints, etc., etc., that may be presumed to have depended upon hereditary syphilitic taint, there have been treated during the past two years 316 cases out of the whole number in 1865 of 1,741 patients, and in 1866 of 1,475 patients."

Of St. Luke's Hospital, Dr. A. A. Davis, resident physician, states "that no venereal cases as such are treated in this hospital; that the diseases, the result of previous venereal diseases, such as iritis, rheumatism, diseases of the skin, bones, and scrofula, etc., about 30 cases are treated annually. The whole number being 934 patients of all diseases annually treated."

Dr. F. Moffatt, physician to the Seaman's Retreat, writes that the whole number treated in the hospital in 1866 was 1,479; venereal diseases, 139; diseases the result of previous venereal infection, 192.

Dr. D. S. Teller, of Mount Senar Hospital, says the regulations of the hospital prohibit the admission of patients with venereal diseases unless they are able to pay for their treatment, except in cases of females, when the disease is communicated by the husband. The number of venereal diseases treated in 1864 was 16; in 1865, 18; in 1866, 22. The number of secondary diseases of a syphilitic character treated in 1864 was 12; in 1865, 12; in 1866, 26. The whole number of patients admitted in 1866 was 569.

From the annual report of the Commissioners of Emigration we find the whole number of patients to have been 5,672; the syphilitic cases, 515.

Dr. W. H. Thompson, who was engaged during the war in examining recruits for the army, replies that the presence of bubo scars on 8,700 men, were in the following percentage rates:

Of the whole number, 8,700, 8 per cent. were scarred; of recruits from the country, 1.05 per cent. were scarred; of 5,000 recruits from the city, 12.25 were scarred. The different nationalities stand in the matter thus: American, 6.75; Irish, 6.08; Germans, 9.08.

To supply the deficient information we have resorted to the published report of Dr. William W. Sanger, resident physician on Blackwell's Island in 1857, who reports as follows: Penitentiary hospital, Blackwell's Island, 2,090; almshouse, 52; workhouse, 56; penitentiary, 430; Bellevue hospital, New York, 768; Nursery hospital, Randall's Island, 734; New

York. State Emigrant Refuge hospital, Ward's Island, 559; New York Hospital, Broadway, 405; New York dispensary, 1,580; Northern dispensary, 327; Eastern dispensary, 630; Demilt dispensary, 803; Northwestern dispensary, 304; Medical college, 207; King's County hospital, 311; Brooklyn City hospital, 186; Seaman s Retreat, 365. Total, 9,847 cases of venereal disease; and if we add to this an equal number for the cases attended in private practice, which (considering the multitude of such persons attended by the regular physicians and the advertising quacks) is probably less than the truth, we shall have 19,694 cases of venereal disease in the Metropolitan health district.

Mr. Kennedy, Superintendent of Police, thinks that about 15 per cent. of the men who present themselves for examination to serve as policemen are excluded on account of venereal diseases.

The fountain of these diseases must be sought for among the prostitutes; and from the report made, with great accuracy, by Mr. Kennedy, we find the houses of prostitution are 569; houses of assignation, 90. The whole number of prostitutes, that is, of women who pursue prostitution as a means of livelihood, and not including those who practice private debauchery, amounts to 2,574.

To speak of the prostitution and the diseases resulting from it, and especially to propose to treat these diseases and subject the wretched women who pursue this occupation to police and medical surveillance, in hopes of eradicating these diseases, is at first sight revolting, and has always met with opposition, even in those countries which ultimately have changed their views upon the subject. Many thoughtful and good men have in times past urged upon the authorities the necessity of this control; but no one can review the subject without wounding the sensibilities of many worthy persons, who believe that to undertake to treat prostitutes would be to give encouragement to vice.

Virtue is the result of natural disposition, or arises from education and early training, which makes the moral nature elevated, conscientious, and religious, and not the outgrowth of the dangers incurred from debauchery.

Since the moral view is likely to be confounded at every step, it is proper to give to it thoughtful consideration.

If it were proposed to establish prostitution in a place where it had never existed there would be a united and emphatic protest against such a measure. But since prostitution has always existed in all large communities, and the whippings inflicted in times past upon the diseased of both sexes in entering and on leaving the hospitals, the denunciations of the church, the penalties imposed by the law, in addition to the dangers of disease and loss of character, have all been powerless to prevent it, what shall be done? Are we to allow the diseases thus generated to go on to destroy the people? If debauched people lead infamous lives, are we to say that they and their companions in vice are suffering the just penalty of their profligacy? that they have offended against the laws of God, and ought to be left to suffer from ruined health, and to give disease to the innocent? Censure, when carried to cruelty, cannot be attended by any good result. It may make the offender defiant, or fill him with despair, but it is not likely to lead to his reformation. Such stern views of duty cannot be held for any length of time; for most of the diseases which afflict mankind arise from some infringement of the law of God. When one is prompted to be harsh in his judgment of others let him reflect how slight a step in many instances exists between the evil thought and the real act, that the circumstances of time and place, early associations and teachings of a colder temperament, may have been the only restraints that have delayed and saved the tempted. Let him also reflect that if the Creator has affixed penalties to vices, He has in his wisdom and goodness furnished us with remedies to treat the diseases which follow from them, and if we are wise and thankful we shall not hesitate to use them. Physicians who are most familiar with diseases and their origin, and who have fixed and elevated views of the obligations of their profession, are not likely to allow the miserable to suffer if they have the means of relieving them, and will not allow abstract questions of casualty to perplex their judgment or to lead them to inquire how the disease was contracted when the sufferer is appealing to them for help.

The municipal authorities have spent large sums for hospitals, and many thousands of dollars are employed annually for the treatment of venereal diseases, and as the population of the district grows so must the hospital accommodations be increased; whereas, if they had treated these diseases from their origin, and had endeavored to *prevent* them, the results would have been greater, and the pecuniary outlay very much less. They have acted as if they believed that public censure would attach to them if they treated prostitutes efficiently.

Some of the dispensaries and hospitals which receive large contributions to their funds from the State and cities, have proposed to charge those affected with syphilis a certain sum for medicines, or in some way make distinctions between them and the other patients. The managers, it would seem, relieve their minds by such conduct from all responsibility in the matter; as if a penalty or a fine would make that right which was immoral in itself; as if their narrow notions could impose rules of conduct on the physicians who would regard them as impertinent, and consequently to be disobeyed; as if the public had no interest in having contagious diseases cured, and would patiently continue their contributions to the support of these institutions, while both guilty and innocent were held up to ignominy, and the peace of families destroyed by the exposure of their illnesses, which such distinctions would cause.

It is time that we had made up our minds on this subject, and if it is immoral to treat prostitutes with venereal diseases, let us say so at once; let the miserable victims die, and never allude to the subject again. But if it is right to treat them, let us do so efficiently, and not suffer our minds to be swayed by the ever-recurring questions of conscience, which weaken the will and render the execution feeble. We ought to act in this matter with the good sense and resolution we would in any other business, and crush it out in its origin, as we did the cholera.

It is the duty of the Board of Health to take cognizance of everything that endangers the health or lives of the people, and in reply to the Legislature, who have called for information, to say that nothing imperils it more than the prevalence of venereal diseases among all classes. Private individuals may hesitate to volunteer their opinions, but a public body like the Board of Health can properly urge upon the Government of the State the necessity of enactments to give legal authority to measures which are demanded for the eradication of venereal diseases.

For this purpose we would suggest: First, That it shall be the duty of all hospitals and dispensaries in the metropolitan health district which receive pecuniary aid from the State, Cities, or Counties, to receive, treat, prescribe for, and dispense medicines to all persons afflicted with venereal diseases, on the same terms as persons afflicted with other diseases, without any exceptions, distinctions, or charges, founded on the nature of such diseases.

That all keepers of houses of prostitution and assignation shall be registered; so shall all prostitutes who live in these houses; that these registers shall contain their names, ages, nativities, and whether married or not.

These registers shall *not* be open to public inspection. The owners of all houses of prostitution shall be also registered.

In case any woman shall gain admission into a house of prostitution, the fact is to be announced to the public, and the police shall not allow such woman to remain in such house unless she is a registered prostitute.

That the Metropolitan Board of Health shall establish a hospital for prostitutes affected with venereal diseases, and shall appoint physicians for the same, the custody of such hospital to devolve upon the police.

That the Board of Health shall cause to be inspected the houses and persons of all prostitutes, and if any prostitute is found diseased with any venereal affection, shall send her immediately to the hospital.

To carry out these measures, and to provide the means for the support of the hospital, also to establish regulations which shall enable the police to compel obedience in case of resistance, must be left to the wisdom of the Legislature.

By subjecting the owners of houses of prostitution to registration, it is believed that it would compel the owners of houses to have regard for the character of their tenants, and prevent the sub-letting of them for infamous purposes. The publicity given to their names would prevent many from renting these houses who now do so with an eye to profit, because it can be done secretly. Registration would also enable the police to close many houses of assignation, which are the schools in which young women begin a career which ends in prostitution. It would prevent, in great measure, the meeting of married women in these houses, for they would fear the watchfulness of the police, and dread the public exposure which might follow.

By inscribing upon the registers the names of the keepers of houses of prostitution, and of the prostitutes, it is probable that their dependence upon the police would be felt by them, and that they would be induced to behave decently without public scandal.

The fear of a long detention in the hospital will compel them to confess their maladies early, and thus not continue their vocation until obliged by aggravated disease to stop.

Thieves and all sorts of malefactors constantly resort to houses of prostitution, and the women there are often thieves themselves. The registration and supervision of them will tend to make them behave honestly and deter them from robbing those who visit them. Being personally known to the police, they can be held responsible for their conduct. Drunken men, and young men just escaped from boyhood, are often found infatuated enough to squander their hard-earned wages of their estates upon these women. The increased facility of detection would serve to prevent these crimes.

Registration will enable parents to trace their children, and in some instances to reclaim them, or in case property is at stake it will enable those concerned to know whether they are living or dead.

The books which contain the names of the registered housekeepers and prostitutes should never be open for public inspection, because the peace of many respectable families would be destroyed by the secret contained in them.

By obliging the housekeepers to report the women who demand admission to their houses, it is believed that the debauching of young girls, often mere children, will be prevented. The police could take charge of them, and also of those women who, having recently fallen from virtue, and who are not utterly ruined, in despair rush to these houses, to embrace the life of a prostitute. The police could arrest them in their career, give them time to reflect, and the Moral Reform society and their kindred institutions could interpose their good offices, restore them to their families, or by gentle words, friendly counsel and protecting care, raise their hopes and lead them to a better life.

The greater freedom from disease, caused by the early medical care given to the prostitutes, might lead the depraved to prefer them to all other women. It may be so, but it would have this good effect, to lessen the demand for those women who frequent the houses of assignation to practise debauchery secretly.

It will be perceived that we have chiefly recommended our measures which seem to be best calculated to eradicate the venereal diseases. These are:

1st—To oblige all hospitals and dispensaries which receive pecuniary aid from the public to treat venereal diseases.

2d—Registration of prostitutes, etc.

3d—Hospitals for prostitutes.

4th—The sanitary inspection of prostitutes.

These, or something suitable as these, must be at the foundation of the proposed reform. The remarks made are intended to be explanatory of their effects. We know that we have not covered the whole subject of control or medical supervision. This must be the work of the legislature. Much must be left to persuasion and to the influence of public sentiment, which we believe is earnest in its demand that prostitution shall be restricted and controlled.

WILLARD PARKER, M.D.,

JOHN O. STONE, M.D.,

JAMES CRANE, M.D.,

} *Sanitary Committee.*

SUPERINTENDENT KENNEDY'S REPORT.

From the reports of the various police captains, made to the superintendent on the 27th of January, the facts relating to prostitution in this city appear as follows:

| | 1867. | 1866. |
|---|---|---|
| Houses of prostitution | 560 | 621 |
| Houses of assignation | 90 | 99 |
| Saloons with waiter girls | 38 | 83 |
| Number of public prostitutes | 2,574 | 2,690 |
| Number of waiter girls | 336 | 747 |

In Brooklyn, there are but six public houses of prostitution, and three of assignation; only fourteen public prostitutes. The difference between New York and Brooklyn is not to be accounted for in the superior virtue of the latter city, but rather in its contiguity to New York, which allows its debauchees to riot here, while their city home is apparently unpolluted.

It will be seen that within the past year there has been a decrease in the number of prostitutes, etc.; but it will tax the credulity of many persons to believe that the figures, as given above, are correct, and still more, perhaps, to hear that in the Eleventh Precinct, which contains 90,000 people, there is not a house of prostitution reported.

Before the war, in January, 1861, there were about 2,100 prostitutes in the city. In January, 1863, there were only 1,600 to 1,700. This diminution was caused by the number of professional prostitutes who followed the army to the war. When the war closed, in 1865, these came back with their numbers considerably reinforced, swelling the figures, as reported in 1866, to 2,690.

